# Cardiac arrhythmia during cold-water immersion: a self-controlled field study with extended rhythm monitoring

**DOI:** 10.1016/j.jesf.2026.200479

**Published:** 2026-04-30

**Authors:** Baptiste Merkt, Cecilia Craviari, Aaron Leigh Baggish, Mehdi Namdar, Philippe Meyer

**Affiliations:** a Faculty of Medicine, University of Geneva, Geneva, Switzerland; b Division of Cardiology, Geneva University Hospitals, Geneva, Switzerland; c Department of Cardiology, Lausanne University Hospital, Rue du Bugnon 46, 1005, Lausanne, Switzerland; d Department of Sports Medicine, Swiss Olympic Medical Center, Rue du Bugnon 46, 1005, Lausanne University Hospital, Lausanne, Switzerland; e Institute of Sports Sciences, University of Lausanne, Synathlon - Quartier Centre, 1015, Lausanne, Switzerland

**Keywords:** Cold-water immersion, Cardiac arrhythmias, Ambulatory cardiac monitoring, Open-water swimming, Recreational athletes, Autonomic nervous system

## Abstract

Cold-water immersion (CWI) is increasingly practiced for its physical and psychological benefits, yet its cardiac safety profile remains poorly defined. This study aimed to assess the arrhythmic burden associated with acute CWI in middle-aged recreational athletes using continuous extended rhythm monitoring. We conducted a self-controlled observational field study during the 2023 Geneva Christmas Cup, the world's largest cold-water swimming event. Twenty middle-aged recreational athletes (mean age 56 ± 6 years; 11 women) underwent baseline cardiovascular assessment and continuous 10-day ambulatory ECG monitoring using a waterproof single-lead device (BodyGuardian Mini®). Thirteen participants (65%) had low-to-moderate and seven (35%) high 10-year cardiovascular risk based on SCORE2. A total of 64 CWI sessions (mean water temperature 7 °C) were detected and analysed. Heart rate increased significantly during CWI, rising by 40 ± 13 bpm (43% ± 16%; p < 0.001). Only one arrhythmic event, a brief non-sustained atrial tachycardia (8 beats at 156 bpm), occurred during immersion. Over the 10-day monitoring period, 17 participants (85%) experienced brief, predominantly benign arrhythmias, with no significant difference in event rates between CWI and non-CWI periods (0.12 vs 0.037 per hour, p = 0.25). No sustained arrhythmias or clinically significant events were observed. In the context of a large, non-competitive cold-water event, CWI was associated with a low arrhythmic burden.

## 1 Introduction

Cold-water immersion (CWI), celebrated for its physical and psychological benefits, is gaining popularity worldwide.^1^ Popular cold-water swimming events, such as the Geneva Christmas Cup, the largest of its kind globally, draw thousands of participants each year.^2^ Although CWI may enhance cardiovascular and immune health, it also induces abrupt autonomic responses that can trigger cardiac arrhythmias.^3^^,^^4^ These include the “cold shock” response and, in some situations, the so-called “autonomic conflict,” both of which have been associated with increased arrhythmogenic risk.^4^^,^^5^ Despite this growing popularity, evidence on arrhythmic risk in middle-aged recreational athletes remains scarce. This study aimed to assess the arrhythmic burden associated with acute CWI in a real-world setting using extended ambulatory rhythm monitoring over 10 days, with each participant serving as their own control by comparing CWI and non-CWI periods.

## 2 Methods

Participants were recreational athletes aged ≥45 years enrolled in the 2023 Geneva Christmas Cup. At enrolment, participants underwent a baseline cardiovascular risk assessment, including a medical interview, physical examination, blood tests, and a 12-lead ECG. The 10-year risk of fatal and non-fatal cardiovascular events was estimated using the SCORE2 tool.^6^ Participants were monitored for 10 days, including the event, using a waterproof wearable cardiac monitor (BodyGuardian Mini®). Continuous single-lead ECG recordings were systematically reviewed, with automated detections and participant-triggered recordings used to aid event identification. Participants kept a daily diary of exercise, including CWI, and arrhythmia-related symptoms. CWI timing was based on participant input and corroborated by heart rate patterns during immersion. Arrhythmia occurrence during CWI and non-CWI periods was compared within participants. All participants provided written informed consent, and the study was approved by the local ethics committee (BASEC number 2023-02058).

## 3 Results

Twenty participants [age 56 ± 6.4 (mean ± SD) years, 11 women] with a mean SCORE2 of 3.9 ± 1.8 completed the study protocol. Thirteen (65%) were classified as having low-to-moderate cardiovascular risk, while seven (35%) were considered high risk according to SCORE2 categories. The average training duration was 5.5 ± 2.9 h per week. Participants performed a mean of 3.2 CWI sessions (median 2.5; range 1–7), for a total of 64 (mean water temperature 7.0 °C). Participants wore the device for an average of 226 ± 32 h, representing 4520 cumulative recording hours, including 8.6 h of CWI.

Heart rate (HR) data during CWI were analysable in 17 participants. In the remaining three, motion artefacts during immersion precluded reliable HR quantification at predefined time points, whereas rhythm interpretation remained feasible based on shorter artefact-free segments. Consistent with previously reported sympathetic activation during CWI,^5^^,^^7^ peak HR increased by 40.6 ± 12.5 bpm, corresponding to a relative increase of 43.5% ± 16% compared with pre-immersion values (p < 0.001).

**Fig. 1:**
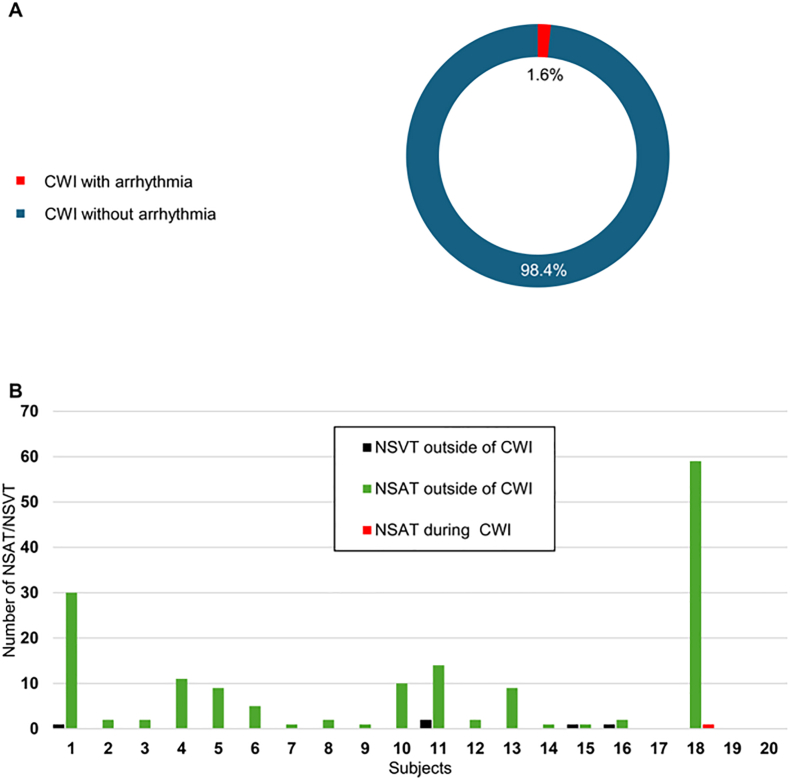
Arrhythmic events during and outside cold-water immersion Panel A: Proportion of CWI sessions with and without arrhythmia. Panel B: Distribution of the 167 recorded arrhythmic events (NSAT and NSVT) across participants, according to their timing during CWI or outside of CWI. CWI, cold-water-immersion; NSAT, non-sustained atrial tachycardia; NSVT, non-sustained ventricular tachycardia.

Only one arrhythmic event occurred during CWI, an 8-beat run of non-sustained atrial tachycardia (NSAT), at 156 bpm (Fig. 1A). Over the 10-day monitoring period, 17 of 20 participants (85%) experienced 167 arrhythmic episodes (Fig. 1B). Events per participant ranged from 0 to 60 (8.35 ± 13.9). Arrhythmias predominantly consisted of NSAT (in 85% of participants) and non-sustained ventricular tachycardia (NSVT, in 20%). Episodes were brief, ranging from 3 to 29 beats. No sustained arrhythmias, atrial fibrillation, atrial flutter, or high-grade conduction disturbances were observed. The incidence rate of arrhythmias was 0.12 per hour during CWI and 0.037 per hour during non-CWI periods (rate ratio 3.1; 95% CI 0.44–22.5; p = 0.25).

## 4 Discussion

The proportion of participants with a documented arrhythmia during CWI (one NSAT episode in 1 of 20 participants, 5%) was low compared to prior studies involving other immersion modalities such as head-out breath-holding (69%) or full breath-hold submersion (up to 92%).^8^^,^^9^ This discrepancy likely reflects physiological differences between immersion techniques and the presence or absence of apnoea. CWI sessions occurred during a mass-participation event in which most participants swam recreationally, without breath-holding and with the head above water. Under these conditions, the risk of ‘autonomic conflict’ is minimized.^4^ Consistent with this, the observed HR increase during CWI reflects acute sympathetic activation. However, pre-immersion HRs were recorded outdoors, where ambient cold may have already induced sympathetic stimulation, potentially leading to an underestimation of the true magnitude of this response.^10^ Together, these findings suggest that the arrhythmogenic potential described in prior studies is not driven by CWI alone, but rather by its combination with apnea or submersion.

Previous studies in patients with established cardiac disease have shown marked increases in ectopy even during mild cold exposure. For example, individuals with chronic heart failure demonstrated a significant increase in ventricular ectopy during 22 °C water immersion.^11^ The lower arrhythmia burden observed in our cohort may be explained by good overall health and regular physical training. Moreover, participants were accustomed to CWI, which induces autonomic habituation that blunts the typical ‘cold shock’ response and may confer protection against sympathetically mediated arrhythmias.^12^

## 5 Limitations

A major strength of this study is the use of continuous 10-day ECG monitoring, enabling direct within-subject comparison of arrhythmia incidence during and outside CWI. Despite the small sample size, multiple CWI sessions per participant enhanced reliability. However, several limitations must be acknowledged. First, some CWI sessions may have been missed or imprecisely marked due to variable participant-triggering of the device, despite the use of diary data to complement timing information; any residual misclassification (e.g., CWI periods classified as non-CWI) would likely lead to an underestimation of the true arrhythmia rate during immersion. Second, head position and breathing technique were not standardised and may have influenced arrhythmia incidence. Third, participants had no known arrhythmogenic structural heart disease, limiting generalizability to populations with established cardiac disease; echocardiography was not systematically performed, and subclinical disease cannot be excluded. Finally, the wide confidence interval (0.44–22.5) indicates that the study cannot exclude either a protective effect or a substantial increase in risk. Based on the observed event rate, detecting a rate ratio of this magnitude with adequate power would likely require ∼200 h of cumulative CWI exposure, corresponding to several hundred participants or substantially greater repeated exposure.

## 6 Conclusion

In this real-world mass-participation context, CWI was associated with a low arrhythmic burden. However, the small sample size and low event rate preclude definitive conclusions regarding safety. Larger studies are needed to better characterise risk.

## Authorship contribution statement

Philippe Meyer conceived and designed the study. Baptiste Merkt and Cecilia Craviari acquired the data. All authors contributed to the analysis and interpretation of the data. Philippe Meyer drafted the manuscript. All authors critically revised the manuscript, approved the final version, and agree to be accountable for all aspects of the work.

## Funding/support statement

This work was supported by the GEcor Foundation. The sponsor had no role in the design of the study, data collection, data analysis, data interpretation, manuscript preparation, or the decision to submit the article for publication.

## Conflict of interest statement for the manuscript

Philippe Meyer reports support for the present work from the GEcor Foundation. The authors declare no conflicts of interest relevant to this article.

## References

[1] Esperland D., de Weerd L., Mercer J.B. (Dec 2022). Health effects of voluntary exposure to cold water - a continuing subject of debate. Int J Circumpolar Health.

[2] Coupe de Noël. https://www.cdn1934.ch/.

[3] Kolettis T.M., Kolettis M.T. (Nov-Dec 2003). Winter swimming: healthy or hazardous?. Evidence and hypotheses. Med Hypotheses.

[4] Shattock M.J., Tipton M.J. (Jul 15 2012). 'Autonomic conflict': a different way to die during cold water immersion?. J Physiol.

[5] Tipton M.J. (Dec 1989). The initial responses to cold-water immersion in man. Clin Sci (Lond).

[6] Visseren F.L.J., Mach F., Smulders Y.M. (2021). 2021 ESC guidelines on cardiovascular disease prevention in clinical practice: developed by the task force for cardiovascular disease prevention in clinical practice with representatives of the European society of cardiology and 12 medical societies with the special contribution of the european association of preventive cardiology (EAPC). Eur Heart J.

[7] Srámek P., Simecková M., Janský L., Savlíková J., Vybíral S. (Mar 2000). Human physiological responses to immersion into water of different temperatures. Eur J Appl Physiol.

[8] Datta A., Tipton M. (Jun 2006). Respiratory responses to cold water immersion: neural pathways, interactions, and clinical consequences awake and asleep. J Appl Physiol (1985).

[9] Tipton M.J., Kelleher P.C., Golden F.S. (Sep 1994). Supraventricular arrhythmias following breath-hold submersions in cold water. Undersea Hyperb Med.

[10] Zenner R.J., De Decker D.E., Clement D.L. (Jan 19 1980). Blood-pressure response to swimming in ice-cold water. Lancet.

[11] Schmid J.P., Morger C., Noveanu M., Binder R.K., Anderegg M., Saner H. (Sep 2009). Haemodynamic and arrhythmic effects of moderately cold (22 degrees C) water immersion and swimming in patients with stable coronary artery disease and heart failure. Eur J Heart Fail.

[12] Tipton M.J., Mekjavic I.B., Eglin C.M. (Sep 2000). Permanence of the habituation of the initial responses to cold-water immersion in humans. Eur J Appl Physiol.

